# Agarose as a Tissue Mimic for the Porcine Heart, Kidney, and Liver: Measurements and a Springpot Model

**DOI:** 10.3390/bioengineering11060589

**Published:** 2024-06-08

**Authors:** Aadarsh Mishra, Robin O. Cleveland

**Affiliations:** Department of Engineering Science, University of Oxford, Parks Road, Oxford OX1 3PJ, UK; aadarsh.mishra@spc.ox.ac.uk

**Keywords:** tissue-mimicking materials, agarose, biomechanics, fractional viscoelasticity, magnetic resonance elastography, ultrasound elastography, acoustic radiation force impulse imaging, rheometry

## Abstract

Agarose gels are often used as a tissue mimic. The goal of this work was to determine the appropriate agarose concentrations that result in mechanical properties that match three different porcine organs. Strain tests were carried out with an amplitude varying from 0.01% to 10% at a frequency of 1 Hz on a range of agarose concentrations and porcine organs. Frequency sweep tests were performed from 0.1 Hz to a maximum of 9.5 Hz at a shear strain amplitude of 0.1% for agarose and porcine organs. In agarose samples, the effect of pre-compression of the samples up to 10% axial strain was considered during frequency sweep tests. The experimental measurements from agarose samples were fit to a fractional order viscoelastic (springpot) model. The model was then used to predict stress relaxation in response to a step strain of 0.1%. The prediction was compared to experimental relaxation data, and the results agreed within 12%. The agarose concentrations (by mass) that gave the best fit were 0.25% for the liver, 0.3% for the kidney, and 0.4% for the heart. At a frequency of 0.1 Hz and a shear strain of 0.1%, the agarose concentrations that best matched the shear storage modulus of the porcine organs were 0.4% agarose for the heart, 0.3% agarose for the kidney, and 0.25% agarose for the liver.

## 1. Introduction

Phantoms or tissue-mimicking materials are used widely in biomedical research for both diagnostic and therapeutic applications [1,2,3,4,5] including computed X-ray tomography [6], elastography [7], single photon emission computed tomography [8], positron emission tomography [9], magnetic resonance imaging [10], ultrasound [11], electrical impedance tomography [12], photocurrent imaging [13], optical imaging and spectroscopy [14], image processing [15], radiotherapy [16], thermal therapy [17], and surgical applications [18]. Biomimetic properties are also important for artificial membranes employed with organ-on-a-chip systems [19,20,21,22] in order to replicate the properties of biological membranes [23,24,25,26,27]. Parameters such as polymer composition, crosslinking density, and surface chemistry can be modified to precisely replicate the properties of particular tissues or organs in phantom form [28,29,30]. Phantoms have the advantage of well-defined and reproducible material properties and so simplify research studies in comparison with real tissue, which is more complex to handle, exhibits greater variability in properties [31,32], and is typically more expensive. Although a range of tissue-mimicking materials has been developed to simulate biological tissues, gel phantoms have been found to represent a wide range of thermal, mechanical, acoustical, and optical properties [31].

This work is motivated by applications where there is both ultrasound propagation through tissue and tissue deformation. Diagnostic ultrasound applications include Ultrasound Elastography (UE), Acoustic Radiation Force Impulse (ARFI) imaging, and Shear Wave Elastography (SWE). In UE, the deformation is created by pressing an ultrasound imaging probe into the body and simultaneously using ultrasound pulses, either A-line or B-mode, to image the tissue deformation and create a map of tissue strain referred to as an elastogram [33,34,35]. In ARFI imaging, the deformation is created by sending a high-amplitude pulse of ultrasound, which results in a volumetric radiation force on the tissue. A-line or B-mode ultrasound imaging is then used to determine the resulting tissue deformation [36,37,38] from which information about the state of the tissue can be extracted. SWE also uses an ultrasound push pulse to generate an acoustic radiation force; however, it then uses very high frame rate (typically greater than 1000 frames/s) B-mode imaging to measure the propagation of shear waves from the ARFI push [39,40,41]. From the shear wave speed, the shear modulus can be estimated; for a linear isotropic material, the shear modulus has a quadratic dependence on the shear wave velocity [36]. SWE has been used in a number of organs including the breast, thyroid, liver, kidney, spleen, pancreas, and lymph nodes [39,40,42,43].

Therapeutic ultrasound applications where tissue deformation can be important include shock wave lithotripsy, burst wave lithotripsy, high-intensity focused ultrasound surgery, and transcranial ultrasound stimulation. In shock wave lithotripsy, high-amplitude shock waves, typically fired at 1–2 Hz, are used to fragment stones [44]. As the shock waves pass through kidney tissue on the way to the stone, the acoustic radiation force deforms the tissue. It has been proposed that cumulative strain develops over the 1000–4000 shock waves required to fragment a stone, which may be a potential mechanism underlying the fatigue and rupture of blood vessels and tubules in the kidney, which is a common side-effect [45,46]. Burst wave lithotripsy is a related emerging technology that employs lower amplitude bursts of ultrasound (100 s of kHz centre frequency), which are used to fragment or push a kidney stone [47]. The bursts of ultrasound are sent at 10–40 Hz repetition frequencies and also have the potential to deform and damage tissue [48]. In high-intensity focused ultrasound [49], high-amplitude ultrasound waves are used to heat and ablate tissue; this also results in tissue deformation that can be used to monitor ablation as tissue stiffens and transitions to an ablated state, which changes the deformation [50,51]. In transcranial ultrasound neurostimulation, ultrasound pulses are used to modulate brain activity [52]. It has been proposed that the stimulation results from the acoustic radiation force activating mechano-sensitive ion channels in neurons [53] and the resulting strain will depend on the mechanical properties of the tissue. In addition, as has been shown in other neurostimulation work, thermal effects [54] can also impact neuronal cells, and it has been proposed that ultrasound can induce both thermal effects and cavitation activity, which may also play a role in ultrasound neurostimulation [55,56]. 

For ultrasound studies, tissue-mimicking materials include agar [31,57], gelan gum [31,58], polyvinyl alcohol [31,59], and polyacrylamide [31,60,61] which have a density and sound speed similar to human tissues and, with the appropriate addition of scatterers, can also match the attenuation and scattering properties [31]. To capture the macroscopic deformation of tissue, the shear modulus is the most important metric [62] in comparison with organoid models, where compressional stiffness and frictional forces are more relevant [32,63,64]. Phantom materials based on agarose are commonly used in ultrasound-based elastography because the acoustic properties match, the elastic moduli can be tuned, and the material is relatively cheap, non-cytotoxic, and easily disposable [65,66]. Agarose is a linear polysaccharide extracted from marine red algae [67], composed of d-galactose and 3,6-anhydro-L-galactose [68] with a melting point at around 85 °C [66]. The basic unit of agarobiose repeats and results in the formation of long polymeric chains. Agarose chains exist in a disordered biopolymer configuration above 40 °C. These chains reorder to form helices on cooling below 40 °C and subsequently aggregate into thick bundles [67,69]. An agarose gel is formed by the development of a three-dimensional network of agarose fibers, formed by agarose helices. This is a kinetically controlled nucleation-based process in which helix–helix aggregation is the rate-limiting step [66,70,71]. The resulting agarose gel structure is a porous solid filled with liquid (i.e., biphasic structure) with both the gel and fluid phases contributing to its viscoelastic property [67,72]. As the water content of the hydrogels is increased, it has a plasticizing effect on the polymer network, which reduces the shear modulus [73] and increases the porosity [74]. This has been reported for the range of agar concentrations employed here using scanning electron microscopy [73,74,75].

Viscoelastic materials exhibit both solid-like and fluid-like properties and possess rate-sensitive stress–strain relationships, implying that the stress–strain relationship changes with the loading rate or the strain rate [76]. Traditional viscoelastic models such as the Kelvin–Voigt model and the Maxwell model use combined springs and dashpots to approximate the mechanical characteristics of viscoelastic materials [76] and have been applied to agarose gels [77]. However, traditional viscoelastic models with a combination of springs and dashpots cannot capture the power-law behaviour of agarose [67]. An alternative to traditional viscoelastic models is the use of fractional order stress–strain elements, which have been shown to describe the viscoelastic properties of multilayered tissue such as cartilage, arterial walls, and cell membranes [78]. A fractional derivative model differs from a classical viscoelastic model in its construction such that components like springs or dashpots are replaced with springpots, which possess intermediate properties between elastic springs and viscous dashpots [78]. Fractional derivative models have been shown to be effective in modelling the response of biological and tissue-like materials that exhibit power-law responses to an applied stress or strain.

In this paper, the viscoelastic mechanical behaviour of agarose gels was characterised using dynamic testing to determine the storage and loss moduli as a function of frequency. The data were fitted to a fractional derivative model and compared to dynamic testing of three porcine organs in order to identify the agarose concentrations that gave mechanical properties that best matched the tissue. It is shown that the fractional models developed here can be used to predict the time domain response of agar when subject to a step in applied shear strain. Finally, when the data from the fractional model are extrapolated to higher frequencies (many kHz), they are consistent with data from the ultrasound elastography literature. This paper is based on work reported in the lead author’s doctoral thesis [79].

## 2. Materials and Methods

### 2.1. Preparation of Agarose Samples

A range of agarose concentrations was prepared for rheometry by mixing 0.25 g to 2 g agarose powder (TopVision R0492, ThermoFischer Scientific, United Kingdom) in 100 mL double-distilled water, resulting in effective concentrations by mass ranging from 0.25% to 2.0%. The mixture was heated in a microwave until boiling was observed, and after removal from the microwave, it was stirred at 240 rpm using a magnetic stirrer. During stirring, the mixture was degassed for 10 min using a vacuum pump to reduce air bubbles. The mixture was then poured into cylindrical moulds of 50 mm diameter and stored at room temperature for 24 h to gel. A cork borer was used to extract agarose samples of a 25 mm diameter. Table 1 lists the sample thicknesses for each of the three rheological tests. The preparation and thickness of porcine organ samples is described in [80]. In addition, samples (*n* = 4) of 0.6%, 1.2%, 1.5%, and 2.0% agarose concentration were prepared only for strain sweep tests with an average thickness of 5.5 mm.

A stress-controlled rheometer (Physica MCR 301, Anton Paar, St Albans, UK) was used to obtain the viscoelastic response in the form of the storage modulus (*G*) and loss modulus (*G*″). The rheometer consists of an upper plate that applies torque to the sample while the lower plate is fixed. The applied torque and angle of deformation is measured and converted to shear stress and strain, respectively. To minimise slippage at the sample–plate interface, sandpaper (200-grit size) was attached to the upper and lower plates. Phosphate-buffered saline was filled in a custom-made metallic casing attached to the bottom plate of rheometer maintained at 20 °C. The samples were pre-compressed up to 10 cycles to achieve a constant force of 0.1 N, after which the measurements were made.

For the uniaxial compression tests, agarose concentrations were prepared with a mass ranging from 0.3% to 2.0%. The preparation was same as the agarose samples prepared for the rheometry tests, and a cork borer was used to extract 25 mm diameter agarose samples of different concentrations and thicknesses, as shown in Table 2. A universal testing machine (Instron 5582) was used to perform the uniaxial tests. A square-shaped perspex plate was attached to the machine crosshead, which compressed the sample at a cross-head velocity of 0.5 mm/min. The applied force F was measured using a 100 N load cell. The displacement δL was measured from the machine crosshead after establishing a contact force of 0.1 N with the sample. The engineering stress was estimated as σ = F/A, where A is the cross-sectional area of the sample, and the engineering strain was estimated as ε = δL/L, where L is the original length of the sample. All agarose samples were compressed until 2% axial strain.

### 2.2. Viscoelasticity

The response of a viscoelastic material is dependent on the strain and the strain rate

(1) $$\sigma =\sigma (\varepsilon ,\;\dot{\varepsilon })$$

where σ is the stress, ε is the strain, and $\dot{\varepsilon }$ is the strain rate. Traditional viscoelastic models use a combination of two classical elements including a spring and a dashpot, to model the viscoelastic response. The spring can be modelled as a linearly elastic material with an elastic modulus (E) where:

(2) σ=Eε

A dashpot can be modelled as a viscous fluid, in which case,

(3) $$\sigma =\;\eta \;\dot{\varepsilon }$$

where η is the viscosity.

**Figure 1:**
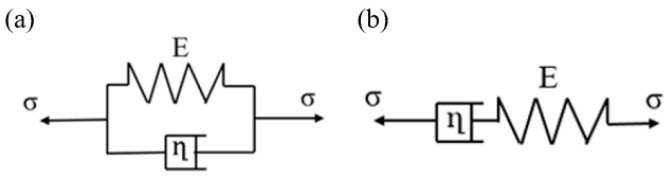
(**a**) The Kelvin–Voigt model and (**b**) the Maxwell model.

Two common models for viscoelasticity are the Kelvin–Voigt model, which consists of a spring and a dashpot in parallel configuration (Figure 1a), and the Maxwell model, which consists of a spring and dashpot in series configuration (Figure 1b).

Fractional derivative elements, referred to as springpots, have been found to better match the viscoelastic properties of soft tissue and gels [67] as they capture the power-law dependence in the frequency domain better than traditional models. The stress–strain relationship for a springpot can be expressed as a fractional order derivative, or as an integer-differential operator

(4) $$\sigma =K_{\alpha }\;\frac{d^{\alpha }\varepsilon (t)}{{dt}^{\alpha }}=\frac{K_{\alpha }}{Ґ\left(1-\alpha \right)}\int_{0}^{t}{\left(t-a\right)}^{-\alpha }\;\frac{d\varepsilon }{da}\;da$$

where 0 ≤ α ≤ 1, *K*_α_ is the coefficient of consistence has units of Pa·(s)^α^, Ґ is the gamma function, and a is an integration variable. The bounding values of α represent the discrete elements employed in conventional viscoelastic models such as a spring *K*_α_ = *G*, when α = 0, and a dashpot *K*_α_ = η, when α = 1. Applying Fourier transform to Equation (4), the storage modulus $G^{\prime }$ and loss modulus $G^{\prime \prime }$ can be written in the frequency domain as:

(5) $$G^{\prime }\left(\omega \right)=K_{\alpha }\;{\left(\omega \right)}^{\alpha }\;cos\left(\frac{\alpha \pi }{2}\right)$$

(6) $$G^{\prime \prime }\left(\omega \right)=K_{\alpha }{\left(\omega \right)}^{\alpha }sin\left(\frac{\alpha \pi }{2}\right)$$

## 3. Results

### 3.1. Strain Sweep Test

**Figure 2:**
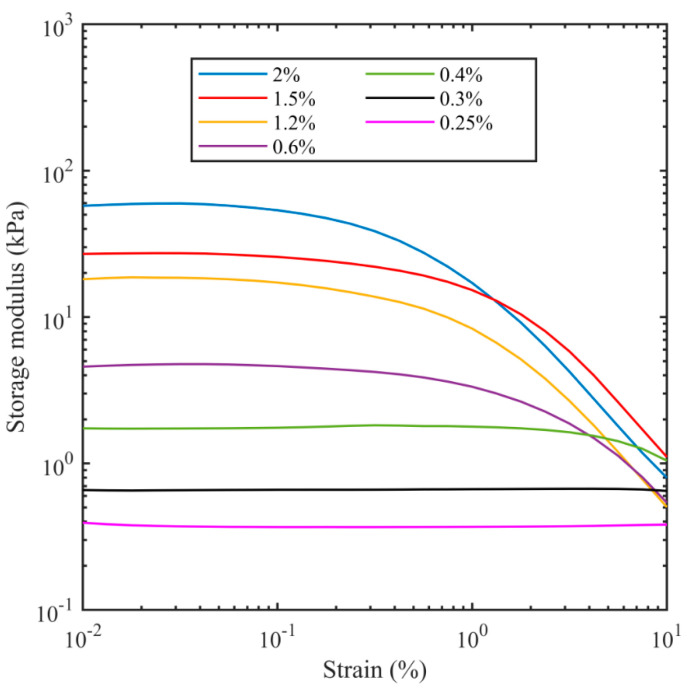
Storage modulus at 1 Hz as a function of shear strain for different agarose concentrations.

**Figure 3:**
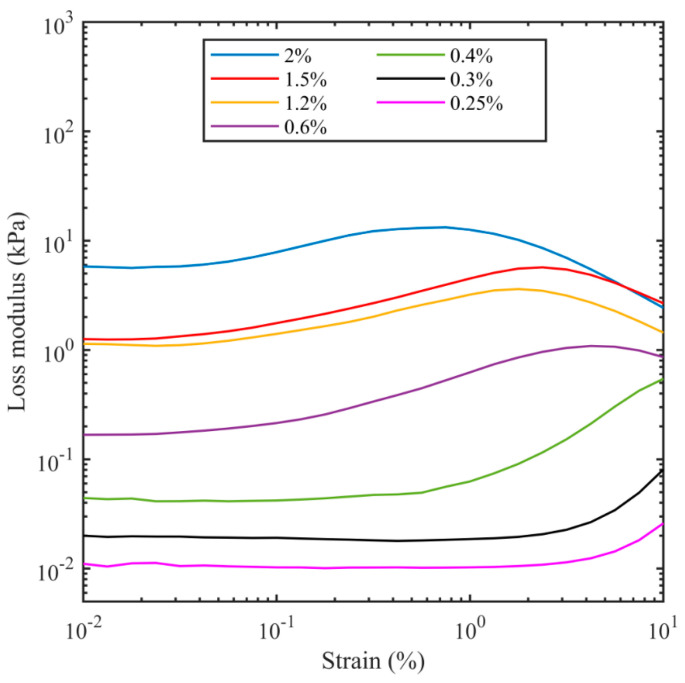
Loss modulus at 1 Hz as a function of shear strain for different agarose concentrations.

The storage modulus and loss modulus were recorded from the rheometer during strain sweep tests as the strain amplitude was increased from 0.01% to 10% at a frequency of 1 Hz. Figure 2 and Figure 3 show the dependency of both moduli as a function of strain for different agarose concentrations. The horizontal sections of both the storage modulus (*G*′) and loss modulus (*G*″) for low values of strain are consistent with the agarose samples acting as a linear viscoelastic material, which implies the moduli are not dependent on the strain amplitude. At higher strains, where the storage modulus drops and the loss modulus increases, the sample no longer acts as a linear material. For the lowest concentration of agar (0.25%), linear behaviour was observed up to strain amplitudes of 10% for the storage modulus and 3% for the loss modulus, but as the agar concentration increased, the linear region decreased such that for 2% agar, it only extended to about 0.03% strain amplitude. This is likely because as the water content decreases, there are more helix–helix interactions, which provide a nonlinearity in the storage modulus of the agarose. The increase in the loss modulus with deformation or strain after linear viscoelastic region is consistent with the existence of an interconnected network of gel particles in agarose formed by cross-linked polymers. As the strain is increased, there are changes in interparticle connectivity [81], resulting in a greater loss modulus.

The effect of the water content on the mechanical properties of the agar gels was also observed with Young modulus measurements in compression. Table 3 shows that the Young modulus decreases as the water content increases; this is consistent with the number of helix–helix interactions that are reduced because of the presence of more water.

**Figure 4:**
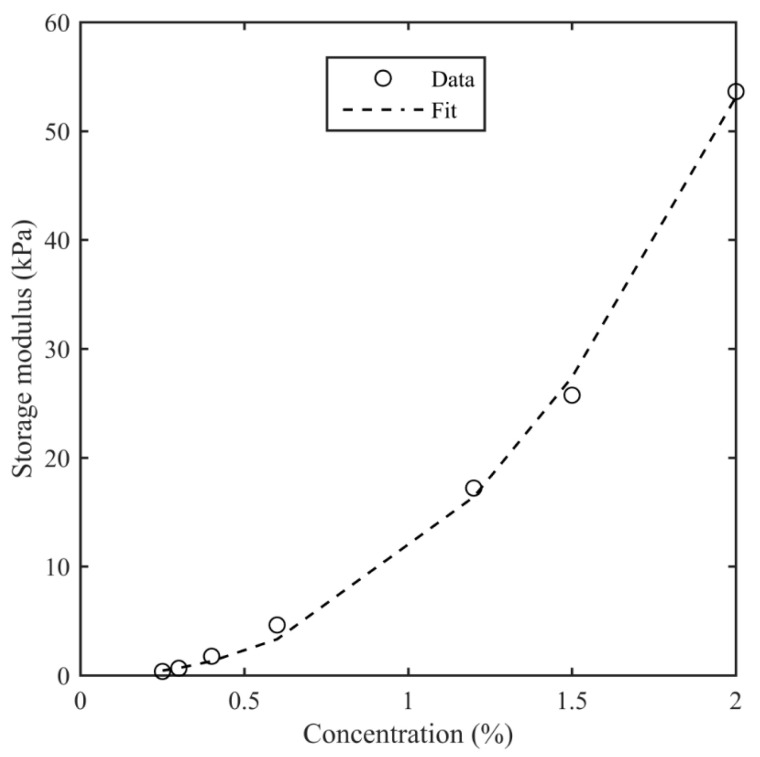
Storage modulus at 0.1% shear strain and 1 Hz frequency as a function of agarose concentration. A power law equation, ${G^{\prime }=Ac}^{b}$, is fitted to the data, where *A* = 10.8 kPa, *b* = 2.3, and *c* is concentration in %.

Figure 4 shows the shear storage modulus at 0.1% strain and 1 Hz as a function of agarose concentrations along with a power law fit. The increase in the storage modulus with agarose concentration (that is, decreasing water content) is consistent with changes in the helical chain arrangement with the agarose concentration. The power law dependence on the agarose concentration has been observed previously [67]. A similar pattern was also observed by reference [81], which suggested that an increase in the storage modulus with concentration or decreased water content is due to an increased particle interaction.

The results from the agar tests were compared to data from porcine organs to determine the best matches for the porcine heart, kidney, and liver. Table 4 lists quantitative values of the storage modulus of the porcine heart, kidney, and liver samples at 0.01% strain and 1 Hz, 0.1% strain and 0.1 Hz, and the properties of the three concentrations of agarose that best matched the porcine organs.

### 3.2. Frequency Sweep

**Figure 5:**
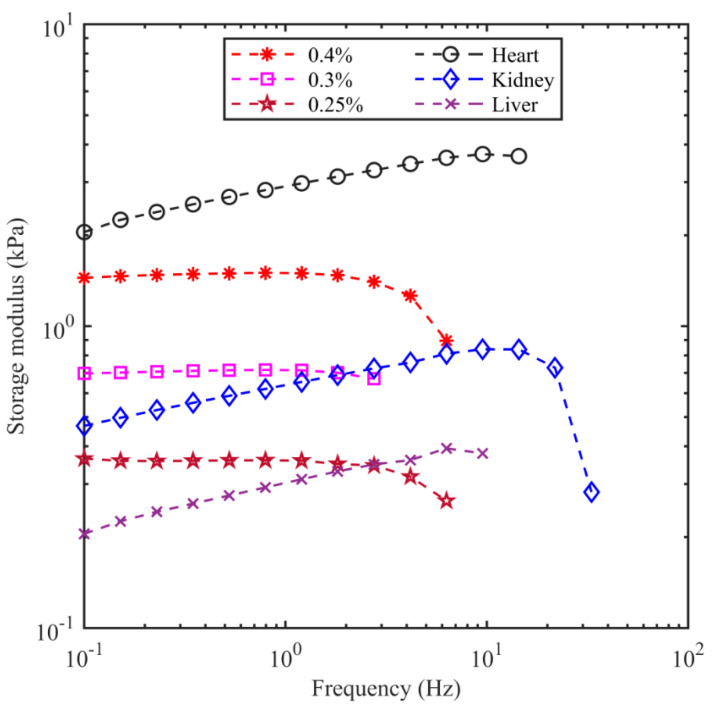
Storage modulus of the porcine organs and agarose samples at an axial contact force of 0.1 N. A drop in the storage modulus can be seen near 10 Hz in the porcine organs and near 1 Hz in the agarose samples, which is indicative of inertial effects.

For the frequency sweep experiments, the strain amplitude was held at 0.1%, and the frequency was increased starting at 0.1 Hz until inertial effects were observed. Figure 5 shows how the measured modulus varies with frequency for the porcine organs and the three best-matching agarose gels. For lower frequencies, the flat or monotonic increase in the modulus is consistent with a viscoelastic medium. The drop in the modulus at higher frequencies signifies the presence of inertial effects on the data, where the rotational inertia of the sample and instrument start to dominate the torque–displacement dependence [82]. Inertial effects occurred at approximately 9.5 Hz for the porcine heart and kidney, 4.2 Hz for the liver, 1.2 Hz for 0.3% agarose, 1.2 Hz for 0.4% agarose, and 0.8 Hz for 0.25% agarose. The measured modulus is not useful above these frequencies because of the contamination by inertia. The quantitative values of the porcine organs and the corresponding agarose concentrations at 0.1% strain and 0.1 Hz are given in the second data column in Table 4.

**Figure 6:**
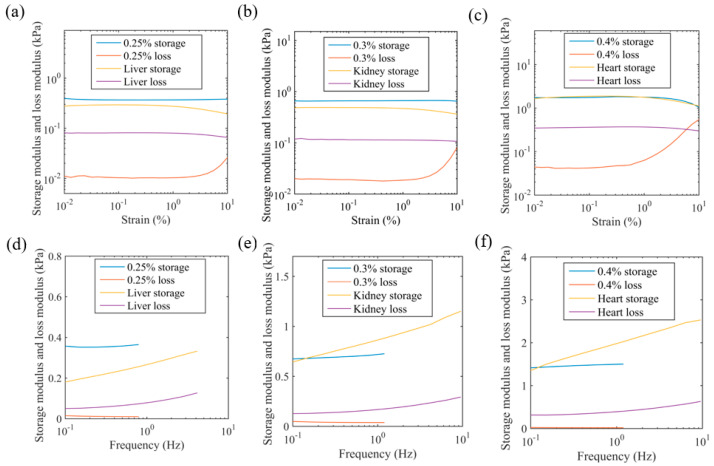
The storage modulus and loss modulus of the agarose and porcine organs as a function of strain (**a**–**c**) and frequency (**d**–**f**). The frequency is 1 Hz for the top row (**a**–**c**), and the shear strain is 0.1% for the lower row (**d**–**f**).

Figure 6 compares the storage modulus and loss modulus of the agarose and porcine organs as a function of strain (top row) and frequency (bottom row). The storage modulus and loss modulus of the agarose and porcine organs remain steady from 0.01% to 0.1% shear strain, suggesting a linear viscoelastic behaviour in this region. There is a monotonic increase in the storage modulus with frequency in 0.4% agarose and 0.3% agarose and a slight reduction followed by an increase in 0.25% agarose. All porcine organ samples show a power law increase in both the storage modulus and loss modulus as a function of frequency. The springpot model (Equations (5) and (6)) was fit to the data using the least square fit function in MATLAB in order to determine *K*_α_ and α. The coefficient of consistence (i.e., *K*_α_) can be interpreted as a material property that measures the binding and unbinding of molecules, and the fractional order (α) is related to the frequency dependence of the material modulus. The values for *K*_α_ and α are shown for all three agarose concentrations in Table 5. It can be seen that the coefficient of consistence in the agarose samples decreases monotonically with their concentration, but there is no change in the fractional order.

Measurements of the shear moduli were repeated over a range of axial strains. It is noted that the strain reported by the testing machine is relative to the initial contact force (F) of 0.1 N, and the nominal stress is given by $\sigma =\frac{F}{A}=204\;Pa$, where *A* is the cross-sectional area of the sample. Uniaxial compressive tests conducted on 0.3% agarose samples (n = 8) using the Universal testing machine yielded an elastic modulus E = 4.4 kPa until 2% axial strain (which is the slope of the average stress–strain curve shown in Figure S4 in the Supplementary Materials). Using E ≈ 4.4 kPa for the agarose samples in $\varepsilon_{A}=\frac{\sigma }{E}$, an axial strain ${(\varepsilon }_{A})$ of approximately 4.6% was then added to all the axial strain values. Although the other agarose samples studied in this paper had concentrations of 0.4% and 0.25%, the offset is likely to be in a similar range.

**Figure 7:**
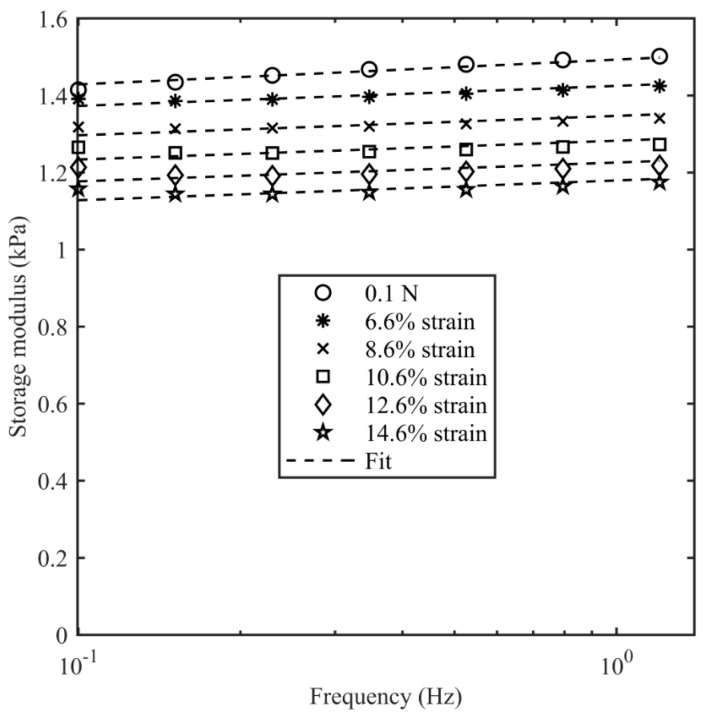
Storage modulus of 0.4% agarose as a function of frequency for 6 different axial strains. A contact force of 0.1 N corresponds ~4.6% axial strain, and the axial strain was varied up to 14.6%. The shear strain is 0.1% at all the values of axial strain.

Figure 7 shows the data and model fit of the storage modulus of the 0.4% agarose samples. It can be seen that there is a good agreement over the range of frequencies and strains with a correlation coefficient better than 0.99. The storage modulus decreased monotonically with the axial strain ($\varepsilon_{A}$) for all agarose samples. The raw data and fitted parameters for the 0.4%, 0.3%, and 0.25% agarose samples are given in the Supplementary Materials as Figures S1–S3, respectively.

**Figure 8:**
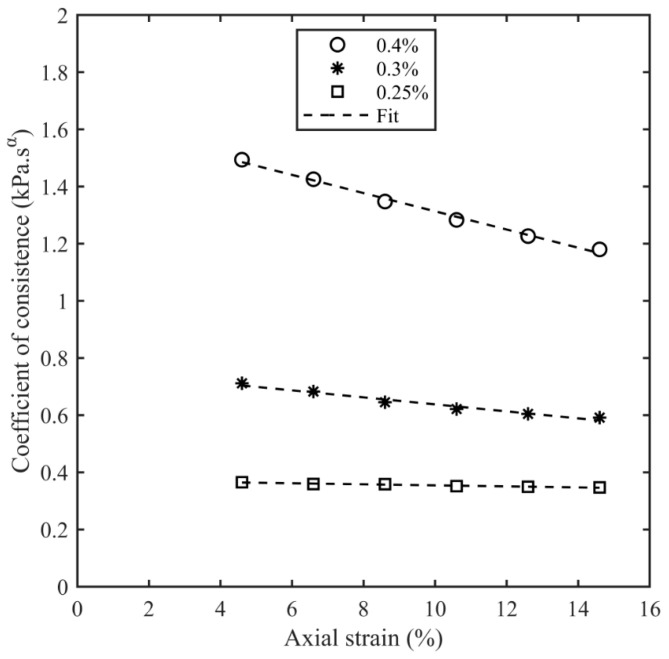
Coefficient of consistence as a function of axial strain for different agarose concentrations.

Figure 8 shows the dependence of *K*_α_ as a function of $\varepsilon_{A}$ for the agarose samples, and a linear relationship with the axial strain is observed. Therefore, the data were fit to the expression:

(7) $$K_{\alpha }=k_{0}(1-b\varepsilon_{A})$$

where $k_{0}$ is the effective $K_{\alpha }$ as $\varepsilon_{A}$ = 0. Parameters for the linear fit of Equation (7) are given in Table 6 for all the agarose samples. It can be observed in Supplementary Materials (Tables S1–S3) that α seems to be independent of the axial strain in the 0.4% and 0.3% agarose samples compared with the 0.25% agarose samples, where it changes with the axial strain.

### 3.3. Stress Relaxation Tests

The fitting of the frequency domain measurements was tested by comparing measurements and predictions of the response of the agarose samples to a step shear strain. The samples were compressed with a 0.1 N load (axial strain of 4.6%), and then at time *t* = 0, they were subject to a step shear strain of 0.1% (within a time scale of 1 s). For a springpot model, the response to a step strain $\in =\in_{0}H\left(t\right)$ (where *H*(*t*) is the Heaviside function) is given by [78]:

(8) $$G\left(t\right)=\frac{\sigma \left(t\right)}{\in_{0}}=k_{\alpha }\frac{\;t^{-\alpha }}{Ґ\left(1-\alpha \right)}$$

where *G* is referred to as the relaxation modulus and Ґ is the gamma function.

**Figure 9:**
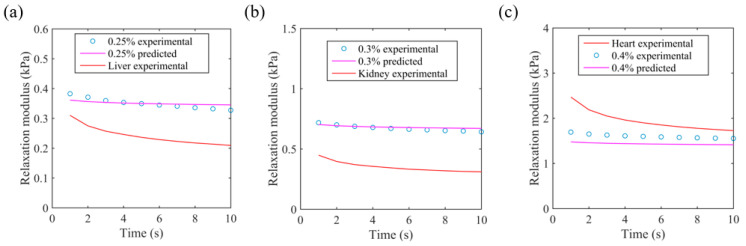
Experimental and predicted relaxation modulus for (**a**) 0.25% agarose and porcine liver, (**b**) 0.3% agarose and porcine kidney and (**c**) 0.4% agarose and porcine heart. The relaxation modulus is in response to a step shear strain of 0.1%.

The measured relaxation modulus was compared to predictions based on the springpot model using the parameters in Table 5. Figure 9 shows the experimental and predicted stress moduli for three different agarose samples along with the data for the porcine organ that they best matched in the dynamic testing. There was generally good agreement between the predicted values and the measured values; the difference was −12% for 0.4% agarose, +1.4% for 0.3% agarose, and +0.3% for 0.25% agarose. It can be seen that the average relaxation modulus of the liver is 31% lower than the 0.25% agarose samples, the kidney is 48% lower than the 0.3% agarose samples, and the heart is 21% higher than the 0.4% agarose samples. The relaxation modulus for the agar samples did not change much over the 10-second time, frame, whereas the tissues all exhibited relaxation on time scales of a few seconds.

## 4. Discussion

The frequency domain measurements of the storage moduli of the agarose and porcine organs are shown in Figure 6. These data show that 0.4% agarose matched heart tissue the best and 0.3% agarose matched kidney tissue the best. It was challenging to match the low stiffness of the liver; a 0.25% agarose phantom exhibited a shear storage modulus 42% higher than the porcine liver at 0.01% strain and 1 Hz. We attempted to prepare agarose samples below this agarose concentration, but the samples could not be tested as there was difficulty in achieving solidification. Frequency sweep tests were performed at 0.1% shear strain, and a springpot model was fitted to the frequency sweep data at different axial strains for all the agarose samples with a correlation coefficient better than 0.99. The coefficient of consistence (*K*_α_) decreased monotonically with the axial strain for all the agarose samples. This damping effect is potentially due to the lower concentrations of agarose tested in this paper. At lower concentrations, helical chains form clusters, whereas at higher concentrations, the chain network spans across the entire sample [79,83,84]. Reference [85] performed viscoelastic testing on freshly harvested porcine hearts. The storage modulus measured by reference [85] ranged from 20 kPa at 0.5 Hz to 30 kPa at 3.5 Hz, and the loss modulus ranged from 3 kPa at 0.5 Hz to 6 kPa at 3.5 Hz. Reference [86] performed a Warner–Bratzler shear test on lamb organs and measured the storage modulus as approx. 15 kPa and the loss modulus as approx. 3 kPa for the heart. This study also obtained a kidney tissue storage and loss modulus of 2.38 ± 0.43 kPa and 0.40 ± 0.08 kPa, respectively, and a liver tissue storage and loss modulus of approx. 1 kPa and 0.25 kPa, respectively. Reference [86] also tested agarose samples and obtained a storage modulus of 2% wt. agarose as approx. 20 kPa. Considering the variability in mechanical properties of animal organs, the storage modulus and loss modulus values obtained in our studies for the porcine heart, kidney, and liver are consistent with the other studies performed on animal organs. The predictive power of the springpot model was tested by comparing the predicted time response to a step shear strain with the measured response. The curves were qualitatively similar to the stress relaxation amplitudes of the predictions and measurements within 12%. It was observed that the relaxation time of the modulus of the agarose samples was much longer than the porcine organs. This is consistent with the storage modulus of the agar samples having a weaker dependence on frequency than the porcine organs.

Although previous studies have developed phantoms of organs such as the liver [87], heart [88], and kidney [89], the accuracy of viscoelastic models in tissues and phantoms has not been quantified in the literature, which is a novel feature of our study. Secondly, all the samples here were measured using the same equipment and procedure, which removed a source of variability that can occur between different studies. A third novel factor is that constitutive models fitted to frequency domain data were used to model the time domain response, and the predicted response was quantitatively compared to the experimental results in the time domain. This study focused on matching the shear modulus of porcine tissue. Other properties such as porosity, nutrient transport, cellular behaviour, and biological compatibility, which are important for other applications, e.g., organ-mimetic hydrogel scaffolds [90,91], were not matched, but the doping of agarose can be performed with other biomaterials [90,91].

In this study, we also looked at the effect of the environment on the mechanical properties by immersing the agarose and kidney samples in phosphate-buffered saline (PBS) and increasing the temperature to 37 °C using a peltier plate in the rheometer. The PBS maintains a pH of around 7.4, similar to tissue, and 37 °C is the nominal body temperature. The shear modulus of the 0.4%, 0.3%, and 0.25% agarose samples and the porcine kidney at 0.1 Hz varied by less than 10% as the temperature was increased from 20 °C to 37 °C (see Tables S4 and S5 in the Supplementary Materials). In future work, changing the PBS concentration to determine the effect of pH changes on mechanical properties could be considered. The effect of ageing on the agar samples was not considered here. Previous studies suggest that agar gels remain stable for a few days; for example, Ref. [92] reported mechanical properties remain stable for five days, and Ref. [69] showed that the Young modulus decreases over time with a drop of 10% at 3 days and 15% at 7 days. These data suggest that the effect of ageing should be considered if studies take place over many days.

The identification of agarose concentrations that match the kidney, liver, and heart is useful for both diagnostic and therapeutic applications of ultrasound in which tissue deformation is also relevant, such as ultrasound elastography, shock wave lithotripsy, burst wave lithotripsy and high-intensity focused ultrasound. The springpot model can be employed in simulations of the tissue response using finite element modelling (FEM) or some other appropriate model. Other areas where the results reported here might be useful include key-hole surgery, e.g., percutaneous nephrolithotomy, in which a rigid ureteroscope will push on kidney tissue during treatment [93,94], or ultrasound-guided beating heart surgery, where surgical tools are both imaged with ultrasound and pushed against heart structures [95].

## 5. Conclusions

In this paper, the rheological behaviour of agarose gels was studied in both the frequency domain and the time domain for a range of agar concentrations, strain amplitudes, and pre-compression. It was found that the agarose gels were linear for up to 0.1% shear strain. In the linear regime, the frequency response of the agarose gels was well captured with a springpot model. As the samples were put under increasing pre-compression, the shear storage modulus exhibited a monotonic decrease, whereas the loss modulus was not affected. The stress relaxation behaviour of the agarose gels was compared with the relaxation modulus predicted by the fitted springpot model, and the values varied by less than 12%. It was found that the best matches to the porcine organs for the shear storage modulus were 0.4% agarose for the heart, 0.3% agarose for the kidney, and 0.25% agarose for the liver. However, the agarose gel was only able to approximate the viscoelastic behaviour of porcine organs with some features, such as the loss modulus, which was not so well captured. This is likely due to the difference in structural arrangement between the porcine organs (which consist of connective tissues and interstitium) and the agarose gels (with helical chain arrangements). This suggests that developing gels employing more than one material could result in the production of models that capture more properties than the agarose model described here.

## Figures and Tables

**Table 1:** Thickness of agarose samples used for strain sweep, frequency sweep, and relaxation tests.

Agarose Concentration	Thickness [mm]: Strain Sweep	Thickness [mm]: Frequency Sweep	Thickness [mm]: Relaxation Test
0.4% (*n* = 12)	5.4 ± 0.1 [*n* = 4]	5.6 ± 0.3 [*n* = 4]	5.6 ± 0.3 [*n* = 4]
0.3% (*n* = 13)	5.2 ± 0.1 [*n* = 4]	5.7 ± 0.1 [*n* = 5]	5.7 ± 0.1 [*n* = 4]
0.25% (*n* = 11)	5.4 ± 0.1 [*n* = 4]	5.5 ± 0.2 [*n* = 4]	5.2 ± 0.1 [*n* = 3]

**Table 2:** Thickness of agarose samples extracted for uniaxial compression tests.

Agarose Concentration	Sample Thickness [mm]
2% (*n* = 5)	18.1 ± 0.2
1.5% (*n* = 5)	17.0 ± 0.9
1.2% (*n* = 10)	17.2 ± 1.0
0.3% (*n* = 8)	17.2 ± 0.6

**Table 3:** The Young modulus Y for different water/agarose concentrations, demonstrating that Y decreases as the water content increases.

Water	Agarose	Y (kPa)
98%	2%	297 ± 15
98.5%	1.5%	98 ± 2
98.8%	1.2%	49 ± 2
99.7%	0.3%	0.66 ± 0.02

**Table 4:** The storage modulus for different porcine organs and agarose samples under two different testing conditions. The second column consists of modulus values at 0.01% strain and a frequency of 1 Hz, and the third column consists of modulus values obtained at 0.1% strain and a frequency of 0.1 Hz.

Sample	Storage Modulus (kPa)	Storage Modulus (kPa)
Heart	1.9 ± 0.5	1.4 ± 0.5
Kidney	0.5 ± 0.03	0.6 ± 0.2
Liver	0.3 ± 0.08	0.2 ± 0.03
0.4% agarose	1.7 ± 0.06	1.4 ± 0.06
0.3% agarose	0.7 ± 0.02	0.7 ± 0.02
0.25% agarose	0.4 ± 0.03	0.4 ± 0.03

**Table 5:** Springpot model parameters for three agarose concentrations that best matched the porcine organs at a contact force of 0.1 N.

Agar conc.	$K_{\alpha }$ [kPa·(s)^α^]	α
0.4%	1.5 ± 0.06	0.019 ± 0.002
0.3%	0.7 ± 0.02	0.020 ± 0.003
0.25%	0.4 ± 0.02	0.019 ± 0.005

**Table 6:** Linear fit parameters in the plot of *K*_α_ as a function of $\varepsilon_{A}$

Parameter	0.4%	0.3%	0.25%
$k_{0}$ [kPa·(s)^α^]	1.498 ± 0.062	0.709 ± 0.023	0.368 ± 0.013
b	0.021 ± 0.003	0.017 ± 0.003	0.006 ± 0.003
α	0.019 ± 0.002	0.020 ± 0.003	0.019 ± 0.005

## Data Availability

The data are provided in the Supplementary Materials and will also be made available on request to the authors.

## Supplementary Materials

The data presented in this study are available in the Supplementary Materials available online at https://www.mdpi.com/article/10.3390/bioengineering11060589/s1. This paper was adapted from the PhD thesis of Aadarsh Mishra, and the results in this paper have not been published in another journal.
